# Biobased Materials for Skin-Contact Products Promoted by POLYBIOSKIN Project

**DOI:** 10.3390/jfb11040077

**Published:** 2020-10-29

**Authors:** Maria-Beatrice Coltelli, Serena Danti

**Affiliations:** Department of Civil and Industrial Engineering, University of Pisa, 56122 Pisa, Italy; serena.danti@unipi.it

The skin is the body outermost tissue and acts as a barrier and defense line to protect our organs. As such, it is the first one to be in contact with stressing external agents and pathogens. Skin structure and function naturally reflect its physiology, and for this reason skin is provided with high capacity for self-repair [1]. Recently, due to the many (also new) substances that we are exposed to, skin shows hyperreactivity, often leading to dermatitis from irritations and allergies. More than 3000 substances are known to cause dermatitis and the sensitization time after exposure to synthetic or chemically treated materials is decreasing. Moreover, once deeply or largely injured, skin repairing and regenerative capacity cannot be completely sufficient, leading to the need of adjuvant materials. The abovementioned examples suggest why the biomaterial–skin interaction may be crucial in our daily life, when healthy skin is in touch with products for sanitary and cosmetical use, as well as for wound healing (Figure 1).

The European project POLYBIOSKIN [3] is aimed at replacing fossil-based materials with natural origin materials for three relevant skin contact products (sanitary pads/diapers, beauty masks and wound dressings), to enable better biocompatible and eco-friendly options. The project was funded by the Bio-Based Industry Joint Undertaking (BBI JU), which is a public–private partnership between the EU and the Bio-based Industries Consortium. Operating under Horizon 2020, this EU body is driven by the Vision and Strategic Innovation and Research Agenda (SIRA) developed by the industry. The BBI JU is dedicated to realizing the European bioeconomy potential, turning biological residues and wastes into greener everyday products through innovative technologies and biorefineries, which are at the heart of the bioeconomy. BBI’s scope is bridging key sectors, creating new value chains and producing a range of innovative bio-based products to ultimately form a new bio-based community and economy.

In the framework of POLYBIOSKIN activities, several research centers and academies were involved aimed at selecting biobased materials and biomolecules compatible with skin that can show high performances. International collaborations were also activated on these relevant topics. The present Special Issue is thus offering some interesting papers and reviews regarding some strategic scientific objectives that were fundamental for the development of high-performance functional bio-based skin-contact and skin-repair products. Many papers were prepared by researchers involved in this EU project or collaborating with them from a scientific point of view. Other contributes were collected from researchers actively working on this topic around the world.

Several polymers and biomolecules fulfil the criteria of being bio-based, as they are derived from renewable biological resources, e.g., produced from living organisms. These polymers are eco-friendly, biodegradable and often highly biocompatible with the human body. The way these polymers can provide benefits when used in body contact by virtue of their functional properties is a subject of recent research.

Two main classes of bio-based polymers relevant for next generation bio-based industry—biopolyesters, such as poly(lactic acid) and polyhydroxyalkanoates (PHAs), being fully renewable and biodegradable [4,5]—as well as natural polysaccharides—cellulose/starch, pullulan and chitin/chitosan, highly available from biomass or waste food—were mainly investigated for their peculiar properties, such as absorbency and anti-infectivity [6]. In addition, proteins derived from animals (gelatin) and humans (plasma) are also considered for their important biological properties.

Functional biomolecules with specific properties were then considered for the modification of the bio-based substrates. Both incorporation in bio-based composites [7,8] and surface modifications [9] are methodologies that can be applied to bio-based and biocompatible substrates to make them anti-microbial, anti-oxidant or anti-inflammatory. These methodologies, enhancing the high performance of bio-based materials, greatly enhance the competitiveness of bio-based towards fossil-based products. 

Antimicrobial and antioxidant treatments used to date commonly rely on the use of chemicals and vehicles that may result in toxic or inflammatory side effects, allergies, sensitization and irritations. Diaper dermatitis is one of the most common dermatological problems in infancy and in the elderly, but it is also becoming a gynecological problem [10,11]. Such eruptions can be subdivided into primary diaper dermatitis, an acute inflammation of the skin in the diaper area with an ill-defined and multifactorial etiology, and secondary diaper dermatitis, which refers to eruptions in the diaper area with other etiologies. The most important factors in the development of primary diaper dermatitis are due to: (i) water/moisture contact, (ii) friction, (iii) urine, (iv) feces, and (v) microorganisms. Possible treatments so far include the use of disposable diapers, barrier creams, mild topical cortisones, and antifungal agents, which can be insufficient and ineffective. In diapers and sanitary pads, the use of biobased materials on the topsheet and the modification of the surface with functional molecules can be thus fundamental for matching both the production of more healthy and environmentally friendly products. 

Beauty masks are cosmetic products aimed at revitalizing the skin by releasing active substances through the stratum corneum that is active in the enzymatic synthesis of lipids. Indeed, barrier recovery and skin homeostasis are the result of restoration of these lipids, which also involves control and normalization of keratinocyte turnover. Non-woven tissues have been obtained by the gelation method and electrospinning technology, using hyaluronic acid and chitin–lignin microparticles by MAVI [12]. In particular, several possibilities were investigated in POLYBIOSKIN, considering both electrospun bio-based tissues and films obtained by the use of biopolymers like starch and PHAs.

Nonetheless, deep, wide, chronic and acute lesions can challenge skin healing and adjuvant therapies may be needed to restore its function. The injured skin needs to be immediately covered with a dressing capable of restoring tissue integrity, maintaining homeostasis, and preventing invasion of toxic substances and pathogens [13]. Therefore, a medical dressing has to establish a barrier to environmental irritants, impede microbial growth, maintain a moist environment, and allow an exchange of gaseous and nutritive ingredients. Moreover, it should not adhere to the wound, in order to allow new tissue growth, and it must be easily removable. For this purpose, special non-wovens made from engineered biomaterials are used and are biocompatible, non-allergenic, and non-toxic. They also promote wound healing by modulating extracellular matrix (ECM) synthesis and regulating microbial growth. To stimulate self-repair of skin, naturally occurring biomaterials, such as decellularized tissues or tissue-derived biomolecules are usually preferred, often in combination with specific chemical factors. The reasons underlying the use of tissue-derivatives rely on the outstanding biointegration, bioresorption and healing capacity exerted by natural biomaterials in lesion repair. However, important shortcomings are observed, like inter-batch variability and biohazard issues [5,6]. In this context, bioresorbable biomaterials offer their most valuable opportunities, not only as dressings enabling in vivo cell migration, but also, and most interestingly, as scaffolds replicating the tissue morphology with controlled chemical and physical properties. 

Novel tissue engineering approaches exploiting scaffolds made of biopolymers and combined with natural antimicrobial agents offer great potential for skin regeneration, as they provide enhanced safety and reliability, owing to their controllable synthesis and fabrication processes. PHAs have been proven to have very high skin regenerative properties and will be the leading biopolymer used for the novel wound dressing developing POLYBIOSKIN. Finally, advanced analytic methods are important to detect the presence and distribution of these particles and molecules on a biomaterial surface.

Ethically sustainable tests for materials represent one priority of the European Community. Indeed, advanced cellular tools, including specific primary cell models and three dimensional (3D) tissue analogues (first of all, the “skin equivalent”, which has become a widely used standard test in cosmetics) have been revealed to be highly predictive, more consistent and less expensive than animal testing. Although the in vitro tests based on human immortalized keratinocytes, representing the epidermis layer, seem the best option for studying the skin compatibility of products, novel cellular tests based on human mesenchymal stromal cells (hMSCs) can be used to validate the materials for skin-contact, because they can differentiate into fibroblasts to represent the dermis layer. hMSCs play a central role in tissue regeneration and repair, maintenance and turnover as they can differentiate into specific cell phenotypes, and/or mediate other cells’ functions by secreting bioactive factors, known as the trophic function of hMSCs. For this reason, hMSCs are a powerful cellular model to evaluate the influence of new materials for skin that have still yet to be fully exploited [14]. This test, together with cytocompatibility and immunomodulation tests performed on dermal keratinocytes and fibroblasts, can improve and refine the biocompatibility test panel for skin and will serve as a spill-over for fostering the biocompatibility testing in biomedical devices.

By collecting valuable manuscripts for this Special Issue, we aim to focus the attention of the biomaterial and biomedical researchers on the great advantages that bio-based polymers can offer in the wide set of applications dealing with human hygiene, wellbeing and health, which POLYBIOSKIN started to explore, but that still require research and innovation to impact our daily lives.

## Figures and Tables

**Figure 1:**
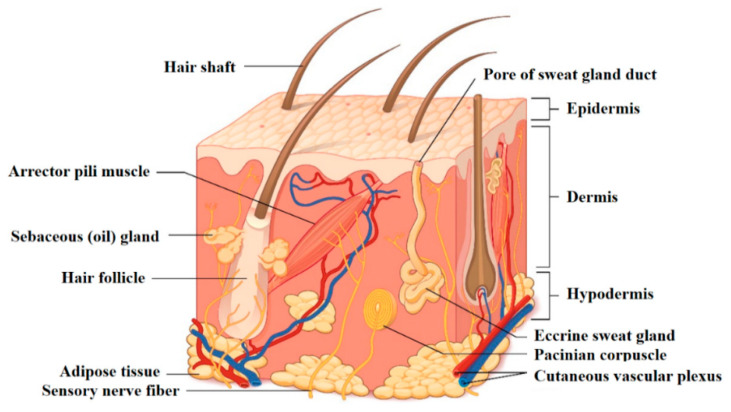
Structure of the skin: a superficial thin layer called epidermis is the first barrier towards the external, provided with cells apt for protection and sensation. Beneath is the dermis layer, which provides both mechanical consistency and nourishing functions; it also hosts sensory receptors and vasculature. The underneath subcutaneous layer is mainly composed of fat for thermic insulation and energy storage. Reprinted with permission from [2] (© 16 January 2020 OpenStax. Textbook content produced by OpenStax is licensed under a Creative Commons Attribution License 4.0 license).
